# Supplementary material for: EnzML: multi-label prediction of enzyme classes using InterPro signatures
Source: BMC Bioinformatics. 2012 Apr 25;13:61. doi: 10.1186/1471-2105-13-61 (PMC3483700; doi:10.1186/1471-2105-13-61)
Supplement: Addtional file 5 — The Java code to format the data files, evaluate and predict. The file enzml_java_code.tar.gz contains the Java code used to format database data to ARFF and XML formats, to execute cross and train-test (jackknife) evaluations and to record evaluation results to database. More information is included in the readme.txt file and the Javadoc files. The code can be used with a MySQL database. To use a different database software, other JDBC drivers might be required. [file 1471-2105-13-61-S5.gz › java_code/enzml2011/doc/uk/ac/ed/inf/enzml/weka/class-use/InstancesFiller.html]

Uses of Class uk.ac.ed.inf.enzml.weka.InstancesFiller


---


|  |  |  |
| --- | --- | --- |
| **Overview** \| **Package** \| **Class** \| **Use** \| **Tree** \| **Deprecated** \| **Index** \| **Help** | |  |
| PREV   NEXT | **FRAMES**    **NO FRAMES**     **All Classes** |  |


---


## **Uses of Class uk.ac.ed.inf.enzml.weka.InstancesFiller**

| Packages that use InstancesFiller | |
| --- | --- |
| **test.weka** |  |
| **uk.ac.ed.inf.enzml.mulan** |  |
| **uk.ac.ed.inf.enzml.weka** |  |

Uses of InstancesFiller in test.weka

| Methods in test.weka that return InstancesFiller | |
| --- | --- |
| `static InstancesFiller` | `InstancesFillerTest.instancesFactory()` |

Uses of InstancesFiller in uk.ac.ed.inf.enzml.mulan

| Subclasses of InstancesFiller in uk.ac.ed.inf.enzml.mulan | |
| --- | --- |
| `class` | `MulanInstancesFiller`             Class |

Uses of InstancesFiller in uk.ac.ed.inf.enzml.weka

| Methods in uk.ac.ed.inf.enzml.weka that return InstancesFiller | |
| --- | --- |
| `InstancesFiller` | `DataSetGenerator.getInstancesFactory()` |

---


|  |  |  |
| --- | --- | --- |
| **Overview** \| **Package** \| **Class** \| **Use** \| **Tree** \| **Deprecated** \| **Index** \| **Help** | |  |
| PREV   NEXT | **FRAMES**    **NO FRAMES**     **All Classes** |  |


---
